# Brucella Endocarditis: A Case Series

**DOI:** 10.7759/cureus.74969

**Published:** 2024-12-02

**Authors:** Sajad A Bhat, Farooq A Guroo, Ajaz N Koul, Suhail Mantoo, Farhana Siraj, Mohammad Yonus Soharwardy

**Affiliations:** 1 Internal Medicine, Division of Infectious Diseases, Sher-I-Kashmir Institute Of Medical Sciences, Srinagar, IND; 2 Internal Medicine, Division of Infectious Diseases, Sher-I-Kashmir Institute of Medical Sciences, Srinagar, IND; 3 Internal Medicine, Sher-I-Kashmir Institute of Medical Sciences, Srinagar, IND; 4 Internal Medicine, Sher-I-Kashmir Institute Of Medical Sciences, Srinagar, IND

**Keywords:** fever, heart failure, human brucellosis, infective endocarditis, septic embolic stroke

## Abstract

Brucellosis is one of the most common zoonotic infections present worldwide. It usually presents as a febrile illness but can affect multiple organs of the body. Although cardiac involvement in brucellosis is rare, it is a fatal organ involvement. The endocarditis is the most common cause of death among patients with Brucella infection. The management of complications of infective endocarditis like stroke requires a multispecialty approach. The Brucella being a fastidious organism requires proper culture technique for growth. The serological methods of diagnosis have limited value in areas with high seroprevalence of brucellosis. We present a case series of three patients who had Brucella endocarditis with varied complications and courses of illness. The practice of a prolonged culture of blood for seven days ensured growth on the culture medium. All three patients were managed with medical therapy only, with a favorable outcome in two patients. In this case series, we have highlighted the importance of proper diagnostic methods and early surgical intervention when indicated.

## Introduction

Brucellosis is an endemic disease in India with a seroprevalence of 11% of the population [1,2]. Lack of proper agricultural precautions and deficient antiseptic practices in rural parts of the country, coupled with a lack of education, leads to a particularly high prevalence of this zoonotic infection [3]. Most of the cases present as non-focal fever, which, when diagnosed early, leads to an uncomplicated course and resolution of the disease. However, severe organ involvement in the form of cardiac and neurological involvement represents another end of the spectrum with high mortality. Cardiac brucellosis can involve the endocardium, myocardium, as well as pericardium; however, endocarditis is the main cause of mortality. The diagnostic modalities of brucellosis include serological testing and cultures. The serological methods of diagnosis have two important disadvantages: first, high seroprevalence in people living in the endemic areas, and second, there is a lag period for the titers of antibodies to rise to a level of significance. The yield of blood culture has been considered to be low and thus not reliable [4]. The management of Brucella endocarditis is controversial, without clear-cut guidelines and a lack of randomized controlled trials. We present three cases of Brucella endocarditis, which were diagnosed by blood culture after a prolonged incubation of seven days. All three patients were managed with medical therapy of combination antibiotics, with a good outcome in two patients.

## Case presentation

Case 1

**Figure 1:**
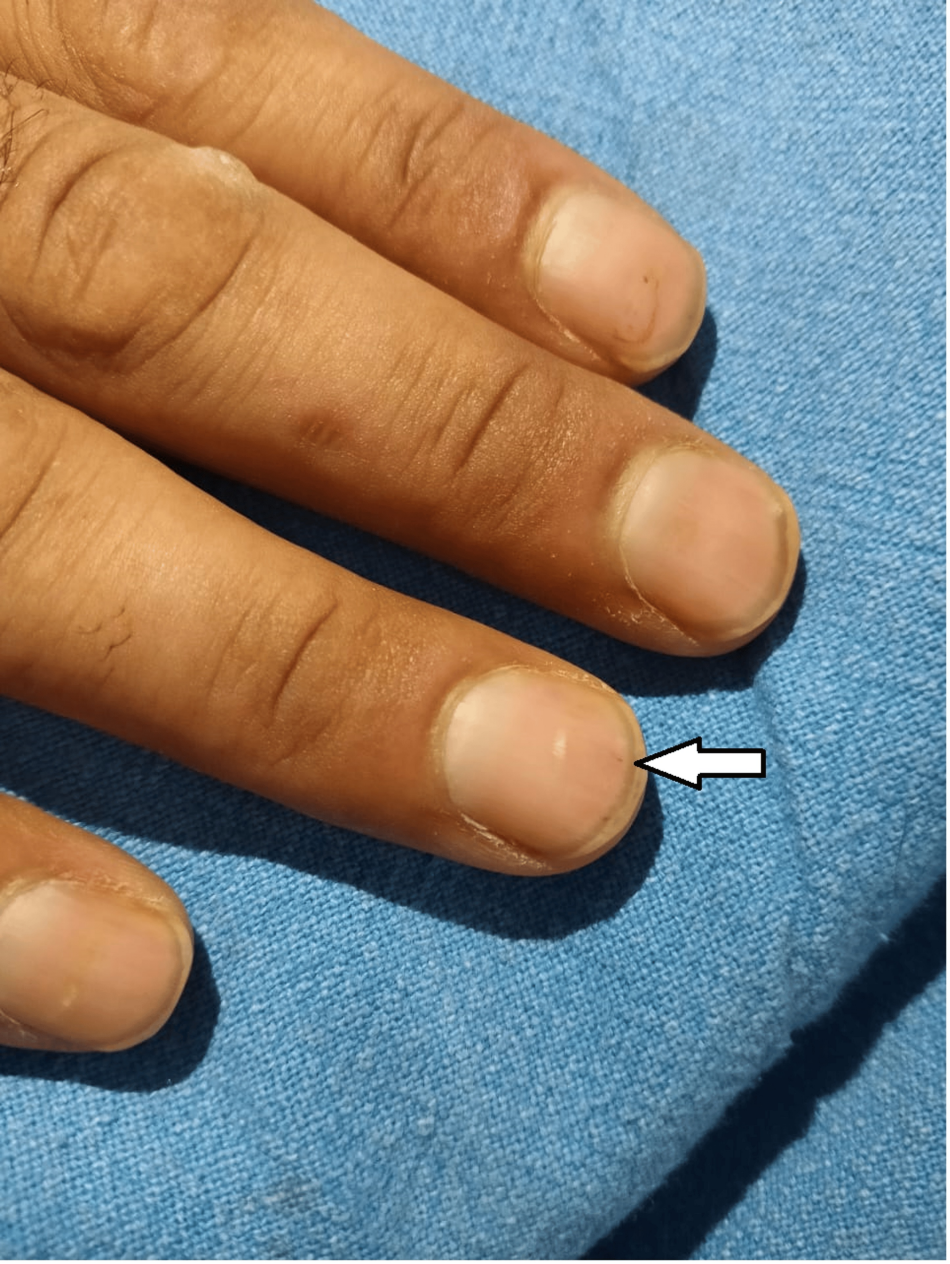
Splinter hemorrhage under the fingernail

A 29-year-old male presented with a one-month history of fever, which was associated with chills, sweating, malaise, and decreased appetite. There was no significant past medical history. On examination, he was febrile with an oral temperature of 102.7 °F. He was tachypnic and had tachycardia. He had a splinter hemorrhage in his nails (Figure 1), and the rest of the systemic examination was unremarkable.

**Figure 2:**
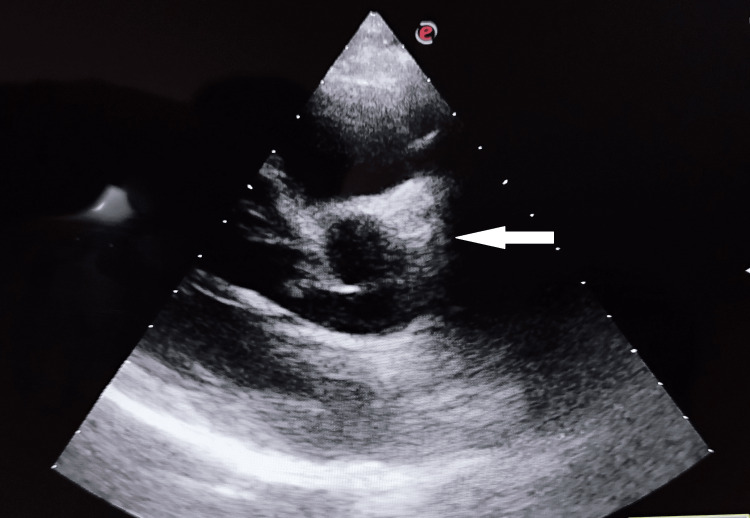
Echocardiography demonstrating vegetation on the aortic valve

The laboratory tests, including a hemogram, kidney function tests, and liver function tests, were normal. C-reactive protein was 29 mg/dl. Out of four blood cultures, one culture showed growth of Brucella melitensis after five days of incubation. Echocardiography revealed shaggy, friable vegetation of the aortic valve along with an aortic root abscess (Figure 2). The standard agglutination test for brucella was positive with a titer of 1: 640.

**Figure 3:**
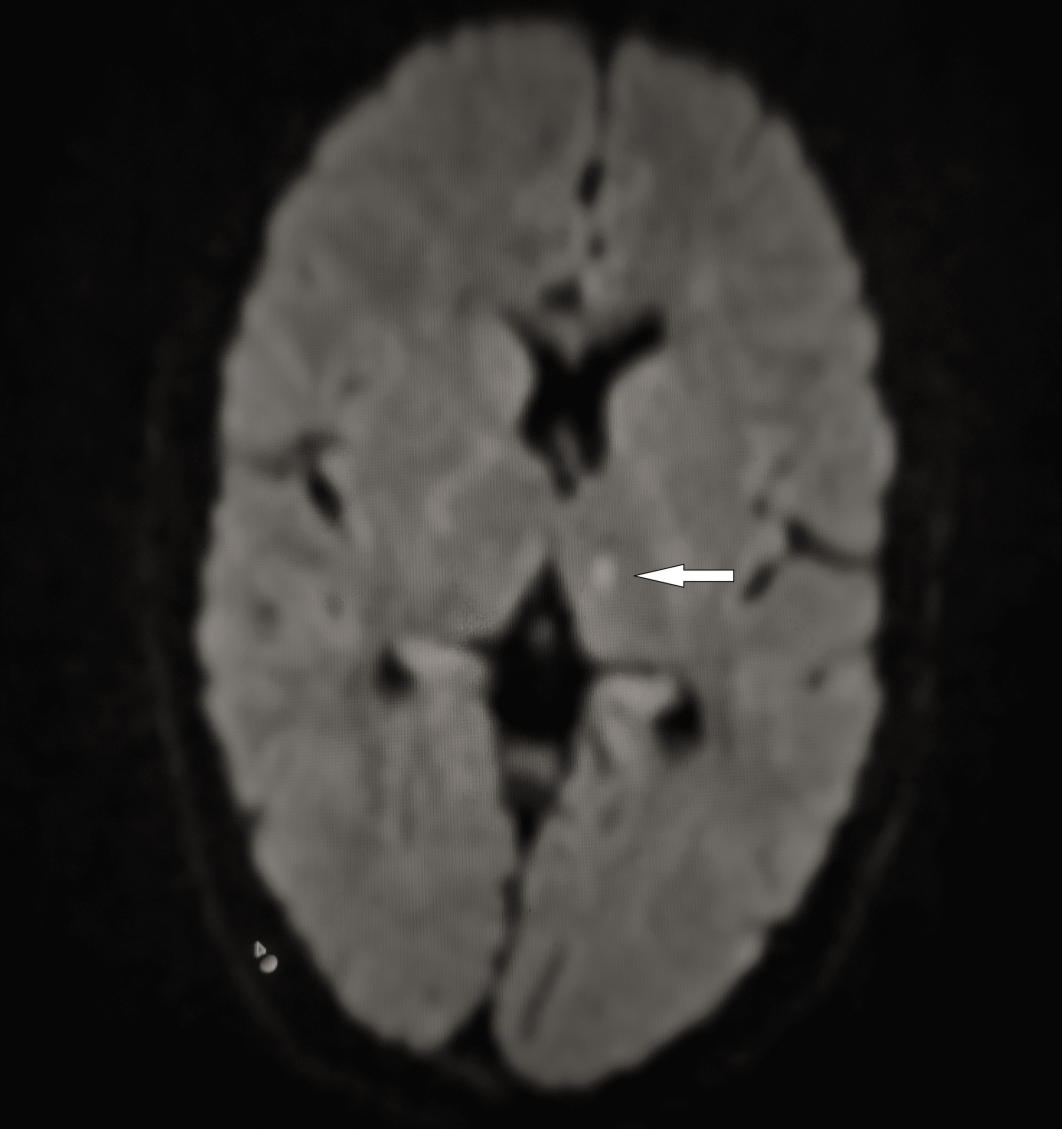
Diffusion-weighted MRI of the brain demonstrating a hyperintense area of restriction in the medial thalamus

The patient was started on triple antibiotic therapy including doxycycline 100 mg twice a day, rifampicin 600 mg daily, and streptomycin one gram intramuscularly daily. After one day, the patient developed a deviation of the angle of the mouth to the right side along with mild weakness of the right hand. A magnetic resonance imaging of the brain was done, which was suggestive of an infarct in the medial thalamus and parietal region (Figure 3).

Injection ceftriaxone two gm intravenously twice a day for six weeks was added in view of embolic infarcts. The patient was planned for a surgical procedure; however, he did not consent to it and preferred medical therapy alone. The patient subsequently developed a rupture of the aortic root abscess into the left ventricular outflow tract. He improved and became afebrile on day seven of treatment and there was no hemodynamic compromise. Medical therapy was continued for a duration of 16 weeks. At the time of cessation of treatment, there was a drop in titer of the standard agglutination test to 1:160, and the patient did not have any signs and symptoms of infection.

Case 2

**Figure 4:**
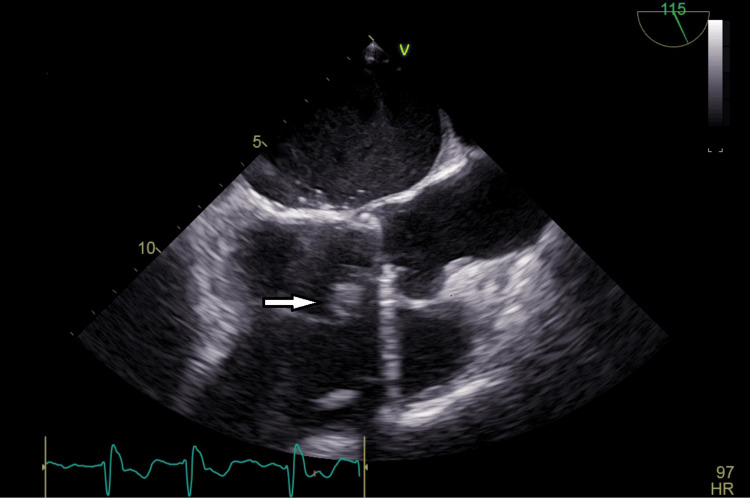
Echocardiography demonstrating a vegetation (white arrow) over the aortic valve

A 46-year-old male, driver by occupation, presented to the emergency medicine department with a history of fever for the last two weeks with chills and rigors. His past medical history was significant for hypertension, diabetes, and rheumatic heart disease. He had undergone a dual valve replacement with mechanical prosthetic valves for the mitral and aortic valves two years ago due to severe mitral stenosis and aortic regurgitation. On examination, he had an irregularly irregular pulse with a pulse rate of 110/min. Systemic examination was normal. Laboratory workup revealed a high C-reactive protein of 33 mg/dl. The electrocardiogram revealed atrial fibrillation. The other baseline investigations, including kidney function tests, liver function tests, and complete blood counts, were normal. Echocardiography showed a 14 x 11 mm vegetation on the left ventricular side of the prosthetic aortic valve (Figure 4).

The four sets of blood cultures were taken and were sterile at 48 hours of incubation. However, one culture grew Brucella melitensis after seven days of culture. The patient was managed with intravenous ceftriaxone, streptomycin, doxycycline, rifampicin, and warfarin. The patient developed a sudden drop in consciousness on day eight of therapy. Computed tomography of the brain revealed an acute subdural hemorrhage, which was attributed to anticoagulation. The patient was managed with a drainage procedure and recovered completely without any difficulty. Therapy was continued for six months and the patient showed complete resolution of symptoms and a drop in titer of the standard agglutination test from 1:320 to 1:80. Anticoagulation with warfarin was restarted after two weeks of surgery.

Case 3

**Figure 5:**
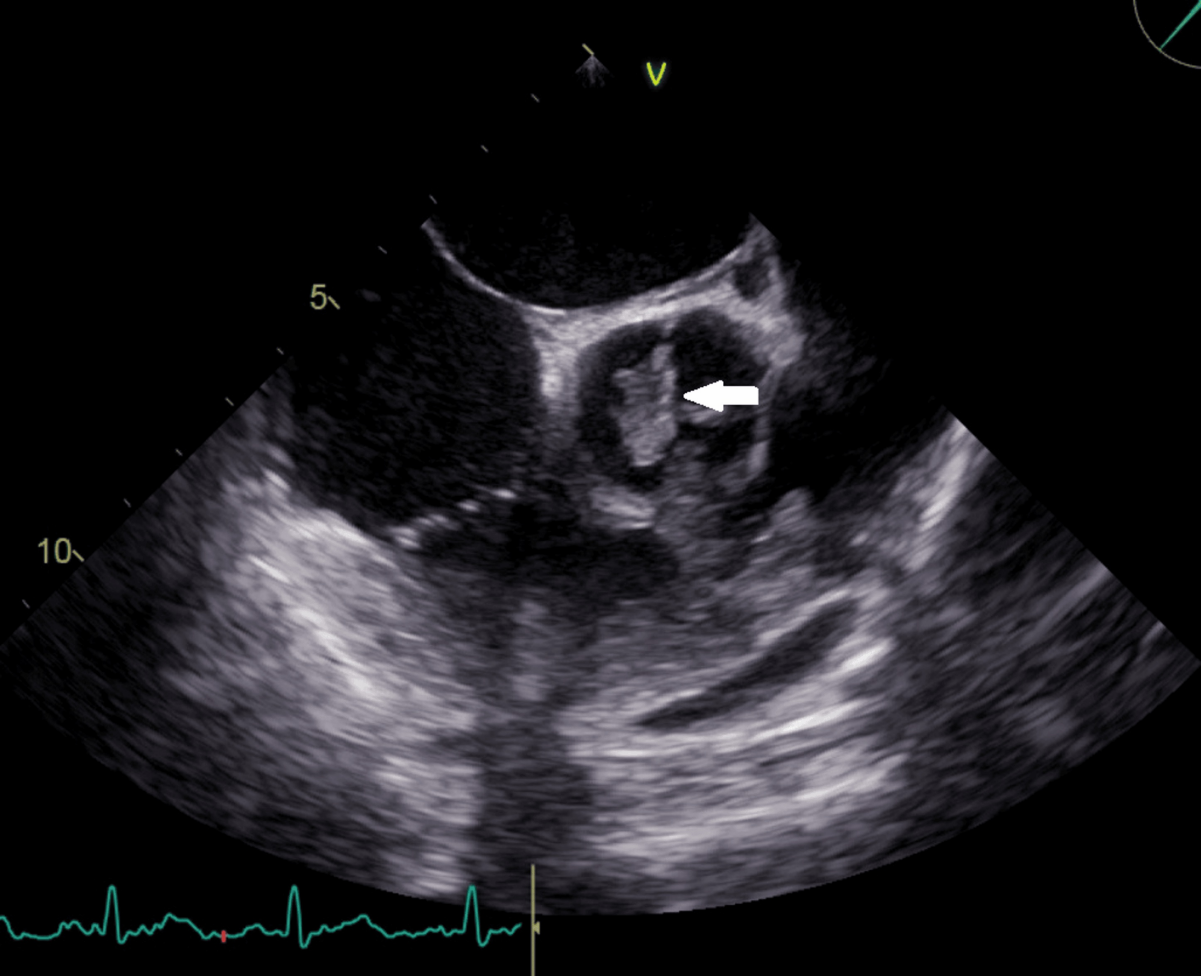
Transesophageal echocardiogram showing a large vegetation (white arrow) over the aortic valve

A 44-year-old female with a medical history of hypertension and previously treated pulmonary tuberculosis was admitted with a two-week history of fever and a one-week history of worsening breathlessness, which was aggravated in the supine position. On physical examination, she had a pulse rate of 140 beats per minute, oral temperature of 101.7 F, blood pressure of 106/60 mmHg, and a respiratory rate of 22 breaths per minute. Her oxygen saturation was 80% on room air, and she exhibited pallor, raised jugular venous pressure, and bilateral symmetrical lower limb pitting edema. Chest auscultation revealed bilateral crepitations at the lung bases, and cardiovascular examination showed a Grade IV pan systolic murmur at the mitral area. Laboratory investigations revealed a normal hemogram with a high erythrocyte sedimentation rate of 48 mm/hour. Renal and liver functions were within normal limits, and urine examination showed no active sediment. Blood cultures taken on three separate occasions were sterile after 72 hours of incubation. A chest X-ray showed bilateral diffuse infiltrates with cardiomegaly. The electrocardiogram revealed normal sinus rhythm. Transthoracic and transesophageal echocardiography demonstrated moderate to severe mitral regurgitation, anterior mitral leaflet prolapse, severe aortic regurgitation, severe tricuspid regurgitation, minimal pericardial effusion, and dilated cardiac chambers with a vegetation on the aortic valve, measuring 9.4 x 7.2 mm (Figure 5).

The patient was initially empirically treated for native aortic valve endocarditis with heart failure using ceftriaxone, vancomycin, and torsemide. The patient was planned for surgery; however, she developed pulmonary edema and was intubated, requiring mechanical ventilation in the intensive care unit. Despite ongoing antibiotic therapy, blood cultures eventually grew Brucella melitensis on the seventh day of incubation. The antibiotics were switched to a regimen of streptomycin, rifampicin, and doxycycline. Over the next 48 hours, the patient developed multiorgan failure, eventually resulting in her death due to shock and cardiac arrest.

## Discussion

Brucellosis causes more than 2.1 million infections per year worldwide [5]. Many species of Brucella have been identified; of them, four are known to cause disease in humans, Brucella melitensis, Brucella abortus, Brucella suis, and Brucella canis. Brucella melitensis is thought to be the most virulent, causes the most severe and acute cases of brucellosis, and is the most prevalent worldwide [6]. The most common presentation is fever associated with night sweats, joint pains, and malaise. Cardiovascular involvement is rare; it includes endocarditis, myocarditis, pericarditis, endarteritis, and mycotic aneurysm of the aorta or ventricles. Endocarditis is the most common cardiac complication seen in 1-2% of cases and the main cause of death in brucellosis, accounting for 80% of mortality in brucellosis infections [7,8].

The three examples described here illustrate the spectrum of brucellosis endocarditis. The first case had native valve endocarditis complicated by an embolic stroke. Stroke is the most prevalent neurological consequence, affecting approximately 35% of cases of infective endocarditis [9]. The use of thrombolysis in infective endocarditis-related ischemic stroke is not recommended due to the substantial risk of bleeding. The majority of patients are treated with antibiotics and surgery. Antibiotics dramatically reduce the incidence of ischemic stroke in infective endocarditis [10]. There is a dearth of data on the use of antiplatelet and anticoagulants in infective endocarditis, making it challenging to manage these individuals. We did not treat the patient with antiplatelet or thrombolysis, and the patient had a good outcome.

The second patient had prosthetic valve brucella endocarditis. This is a very rare occurrence with less than 100 cases reported. This is a particularly difficult case to manage because of the risk of bleeding in patients who require anticoagulation in endocarditis. Taamallah et al. described 51 patients with brucella endocarditis of the prosthetic valve [4]. Brucella melitensis was the most commonly isolated species. The diagnosis was most commonly made with serological testing and blood culture yield was low. In our cases, all three cases were diagnosed based on blood culture. This may be because brucella is endemic in our part of the world, and blood cultures are regularly incubated for more than seven days to detect brucella. The serological tests lose their relevance in high endemicity areas with a high seroprevalence of brucella antibodies. The most frequently affected valve in this report was the aortic valve. 

The mitral valve is the most commonly affected valve in infective endocarditis; however, brucella endocarditis predominantly affects the aortic valve. All three patients in our study had aortic valve involvement, which is in agreement with the literature [11,12].

The management of brucella endocarditis requires a combination of antibiotics and surgical intervention when required. Indications of surgery include failure of medical treatment with uncontrolled infection, congestive heart failure, relapse after recovery by antibiotics, severe hemodynamic instability, and abscess [13]. Case 1 had an aortic root abscess, which was an indication of surgical intervention; however, due to spontaneous rupture into the left ventricular outflow tract, the surgical procedure was deferred and ultimately proved beneficial. The third case who developed acute pulmonary edema suddenly and succumbed within 48 hours did not give time for arranging the surgical procedure which required a multidisciplinary approach.

## Conclusions

Brucella endocarditis should be suspected in individuals who present with signs and symptoms of endocarditis with a history of contact with animals, particularly in endemic regions of the world. Brucella infective endocarditis is a difficult-to-diagnose condition due to the fastidious nature of this bacteria and high seroprevalence in endemic areas. The prolonged culture method of seven days should be regularly practiced in endemic areas to increase the yield of blood cultures. This lethal condition requires prompt medical and surgical intervention for management. In patients with severe valvular dysfunction or features of heart failure, surgical intervention should be performed without delay. The adequate duration of therapy is not known, but at least 12 weeks of combination antibiotics should be prescribed.
